# Supplementary material for: H3K9 methylation regulates heterochromatin silencing through incoherent feedforward loops
Source: Sci Adv. 2024 Jun 26;10(26):eadn4149. doi: 10.1126/sciadv.adn4149 (PMC11204290; doi:10.1126/sciadv.adn4149)
Supplement: Supplementary file 1 — Figs. S1 to S9 Legend for data S1 [file sciadv.adn4149_sm.pdf]

Science Advances
AAAS

Supplementary Materials for

H3K9 methylation regulates heterochromatin silencing through incoherent feedforward loops

Kannosuke Yabe *et al.*

Corresponding author: Soichi Inagaki, soinagak@bs.s.u-tokyo.ac.jp

Sci. Adv. **10**, eadn4149 (2024)
DOI: 10.1126/sciadv.adn4149

The PDF file includes:

Figs. S1 to S9
Legend for data S1

Other Supplementary Material for this manuscript includes the following:

Data S1

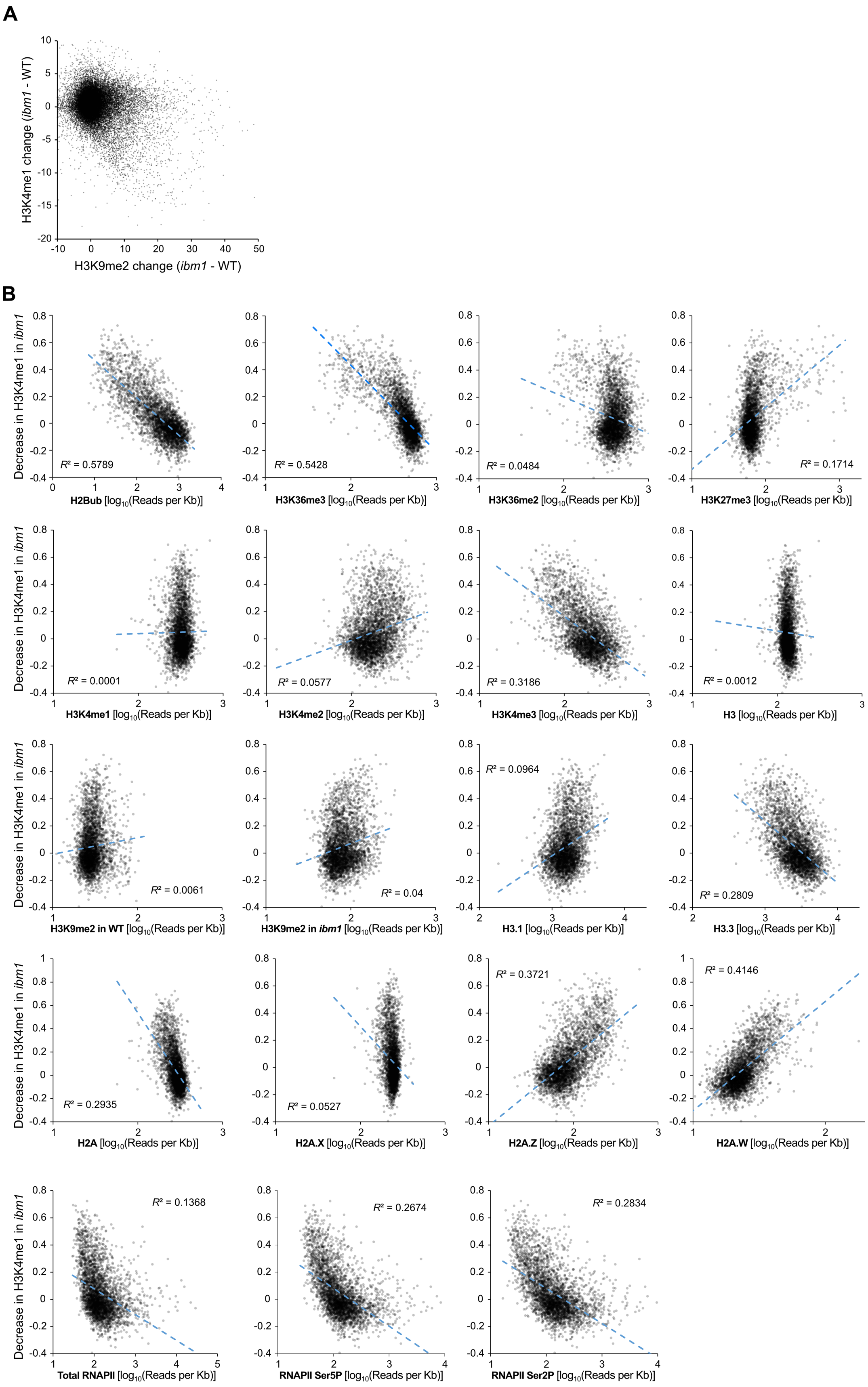
A
H3K4me1 change (ibm1 - WT)
H3K9me2 change (ibm1 - WT)
B
Decrease in H3K4me1 in ibm1
R² = 0.5789
H2Bub [log10(Reads per Kb)]
R² = 0.5428
H3K36me3 [log10(Reads per Kb)]
R² = 0.0484
H3K36me2 [log10(Reads per Kb)]
R² = 0.1714
H3K27me3 [log10(Reads per Kb)]
R² = 0.0001
H3K4me1 [log10(Reads per Kb)]
R² = 0.0577
H3K4me2 [log10(Reads per Kb)]
R² = 0.3186
H3K4me3 [log10(Reads per Kb)]
R² = 0.0012
H3 [log10(Reads per Kb)]
R² = 0.0061
H3K9me2 in WT [log10(Reads per Kb)]
R² = 0.04
H3K9me2 in ibm1 [log10(Reads per Kb)]
R² = 0.0964
H3.1 [log10(Reads per Kb)]
R² = 0.2809
H3.3 [log10(Reads per Kb)]
R² = 0.2935
H2A [log10(Reads per Kb)]
R² = 0.0527
H2A.X [log10(Reads per Kb)]
R² = 0.3721
H2A.Z [log10(Reads per Kb)]
R² = 0.4146
H2A.W [log10(Reads per Kb)]
R² = 0.1368
Total RNAPII [log10(Reads per Kb)]
R² = 0.2674
RNAPII Ser5P [log10(Reads per Kb)]
R² = 0.2834
RNAPII Ser2P [log10(Reads per Kb)]

C

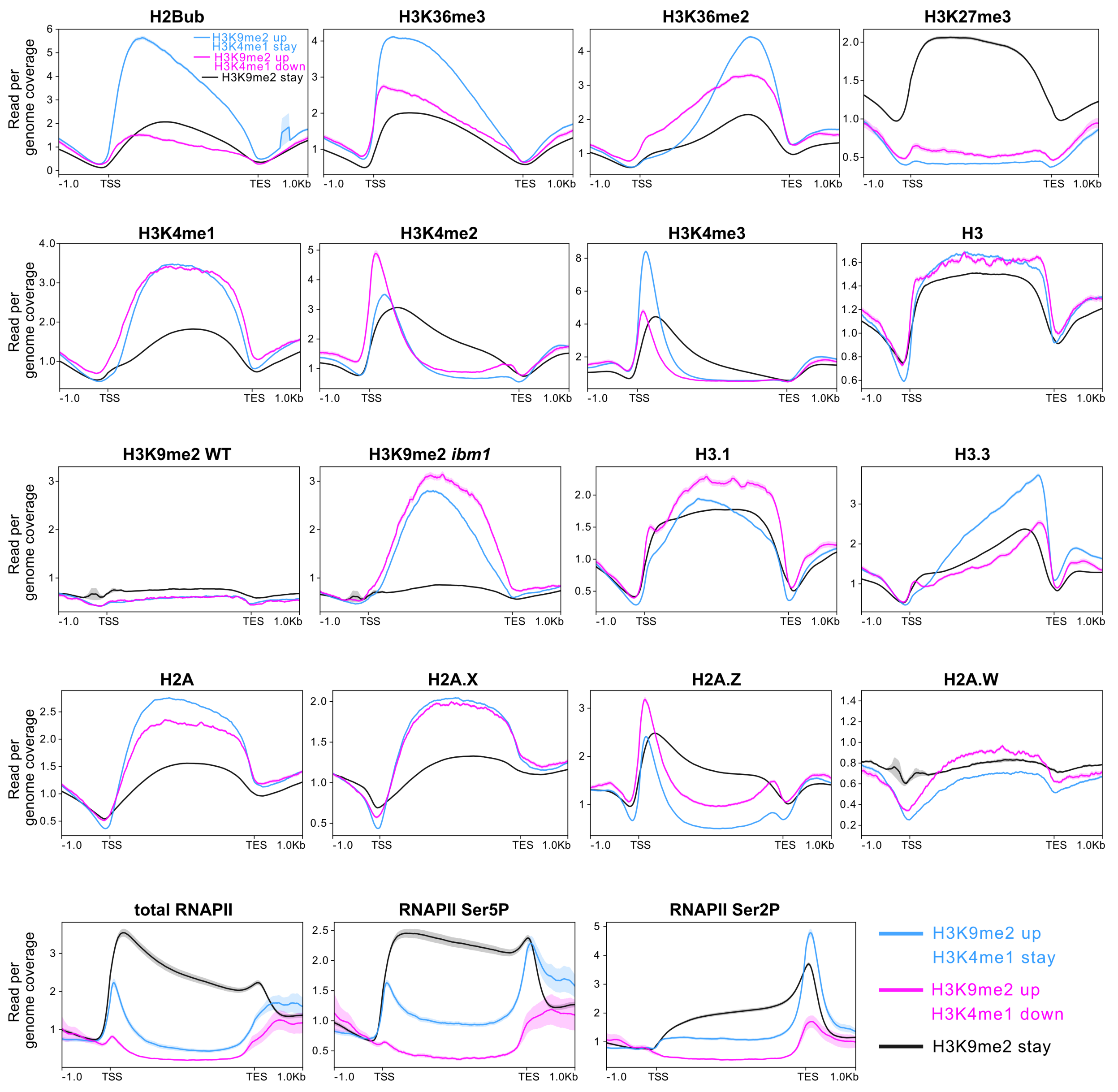

Fig. S1. Screening for silencing modifier(s). (**A**) Relationships between changes in H3K9me2 and H3K4me1 levels (RPKM) within each gene in *ibm1* compared to WT. Data is from ref (*18*). (**B**) Correlations between chromatin features (x-axis), and the decrease of H3K4me1 in *ibm1* (y-axis) are shown as scatter plots, linear regression lines, and Pearson's R^2. Each dot represents each gene that accumulates H3K9me2 in *ibm1*. n = 3,395. (**C**) Intragenic patterns of chromatin features around genes categorized by H3K9me2 and H3K4me1 changes in *ibm1*. Among 3,449 genes that accumulate H3K9me2 in *ibm1* ("H3K9me2 up"), 743 genes showed clear decreases of H3K4me1 ("H3K4me1 down") but others did not ("H3K4me1-stay") (*18*).

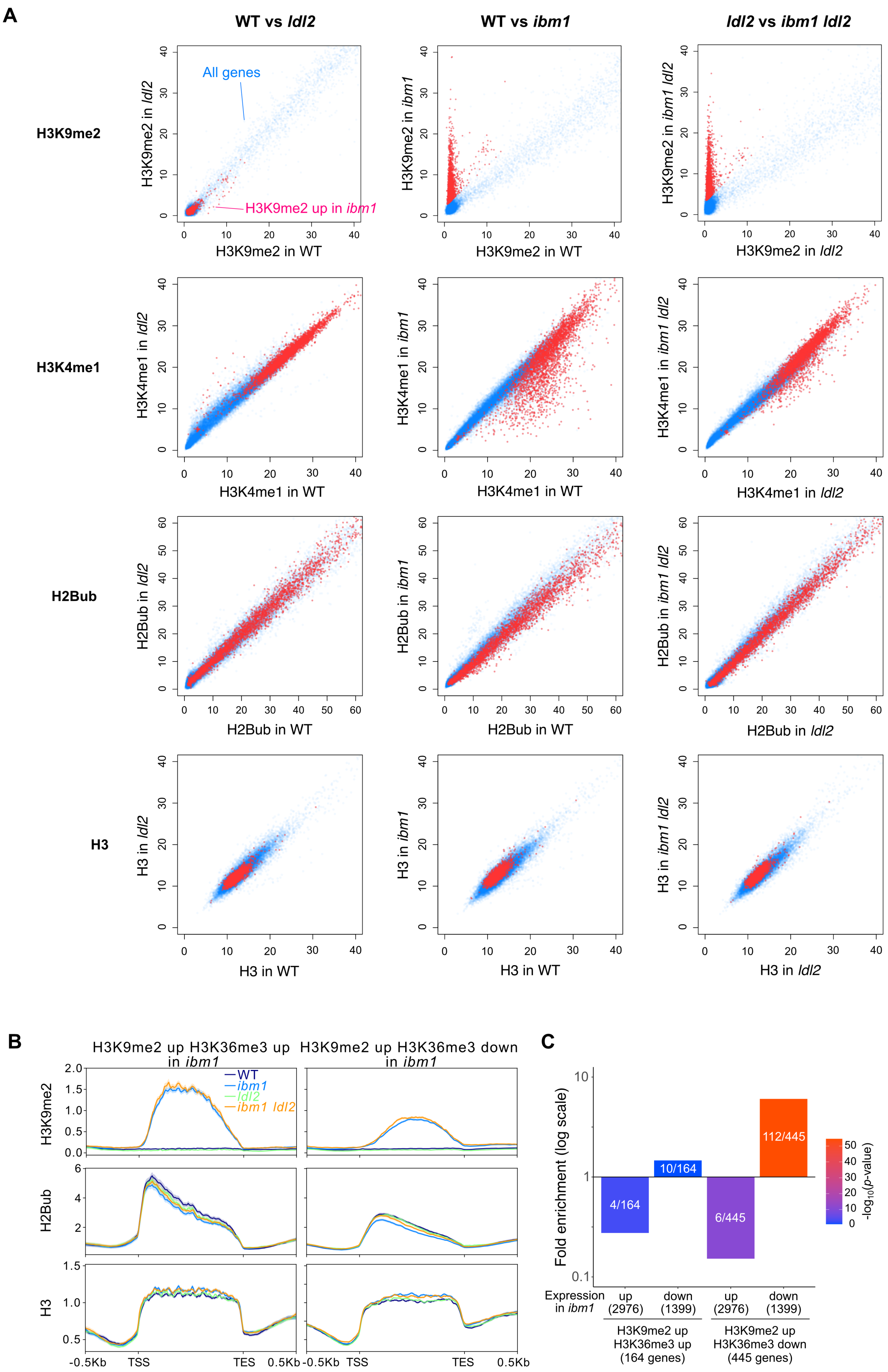
A
WT vs ldl2
WT vs ibm1
ldl2 vs ibm1 ldl2
H3K9me2
H3K4me1
H2Bub
H3
All genes
H3K9me2 up in ibm1
H3K9me2 in ldl2
H3K9me2 in ibm1
H3K9me2 in ibm1 ldl2
H3K9me2 in WT
H3K9me2 in ldl2
H3K4me1 in ldl2
H3K4me1 in ibm1
H3K4me1 in ibm1 ldl2
H3K4me1 in WT
H3K4me1 in ldl2
H2Bub in ldl2
H2Bub in ibm1
H2Bub in ibm1 ldl2
H2Bub in WT
H2Bub in ldl2
H3 in ldl2
H3 in ibm1
H3 in ibm1 ldl2
H3 in WT
H3 in ldl2
B
H3K9me2 up H3K36me3 up in ibm1
H3K9me2 up H3K36me3 down in ibm1
WT
ibm1
ldl2
ibm1 ldl2
H3K9me2
H2Bub
H3
-0.5Kb
TSS
TES
0.5Kb
C
Fold enrichment (log scale)
10
1
0.1
4/164
10/164
6/445
112/445
-log10(p-value)
Expression in ibm1
up (2976)
down (1399)
up (2976)
down (1399)
H3K9me2 up H3K36me3 up (164 genes)
H3K9me2 up H3K36me3 down (445 genes)

Fig. S2. Histone modification patterns in *ibm1* and *ibm1 ldl2*. (**A**) Scatter plots of H3K9me2, H3K4me1, H2Bub, and H3 in *ibm1* and *ldl2* for all genes (blue) and genes accumulating H3K9me2 in *ibm1* (red). The read counts are normalized as RPKM. (**B**) Intragenic patterns of H3K9me2, H2Bub, and H3 around genes categorized by H3K9me2 and H3K36me3 changes in *ibm1*. The y-axis represents the read per genome coverage (RPGC). (**C**) The enrichment analysis of the genes upregulated or downregulated in *ibm1* from ref (*18*), in genes categorized by H3K9me2 and H3K36me3 changes in *ibm1*. The color of the bars is based on the p-value of the hypergeometric test.

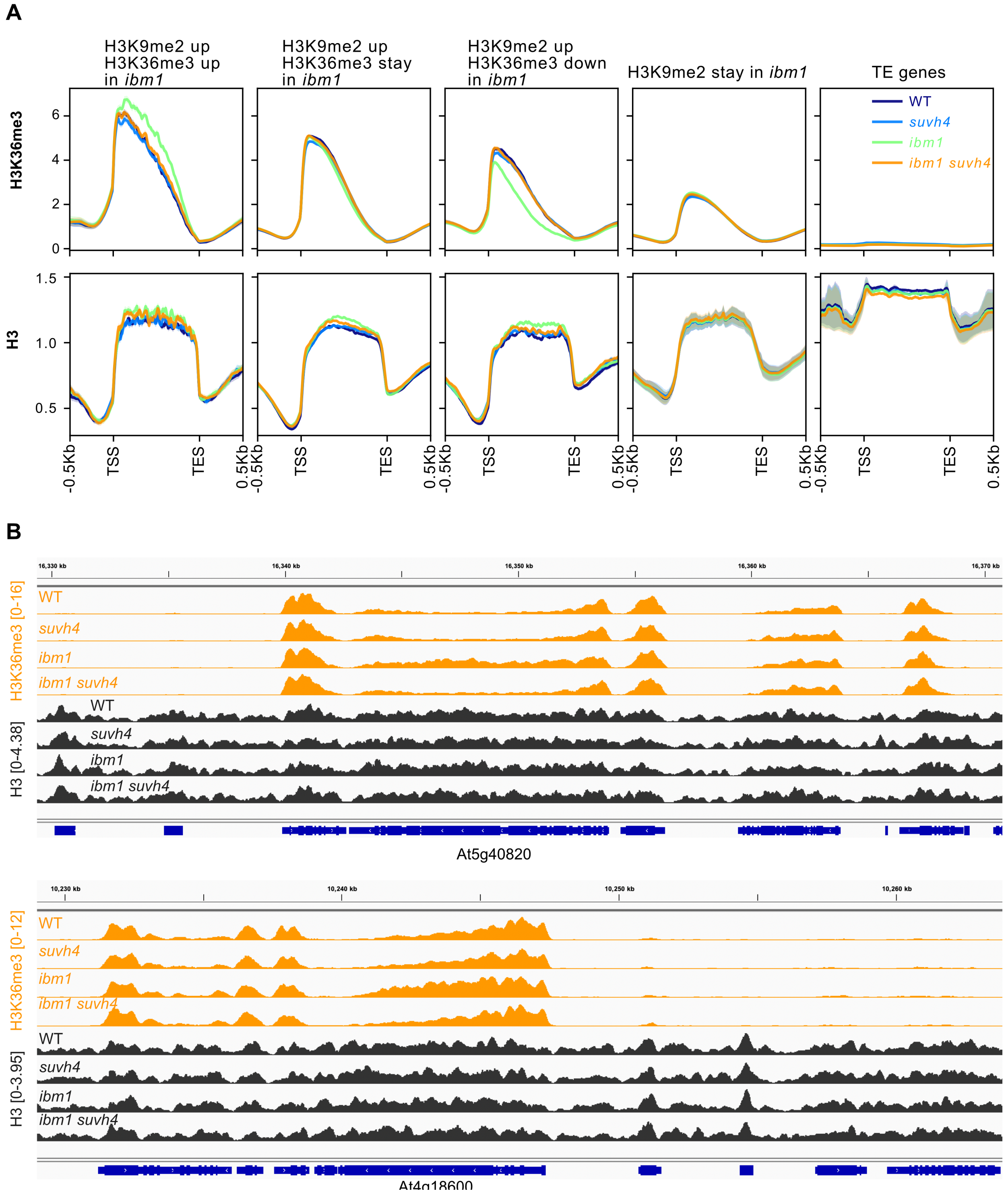

Fig. S3. H3K36me3 pattern in *ibm1 suvh4*. (**A**) Intragenic patterns of H3K36me3 (top) and H3 (bottom) around genes categorized by H3K9me2 and H3K36me3 changes in *ibm1* and TE genes. The y-axis represents the RPGC. (**B**) Browser views around genes with increased H3K36me3 in *ibm1*, which was suppressed in *ibm1 suvh4*.

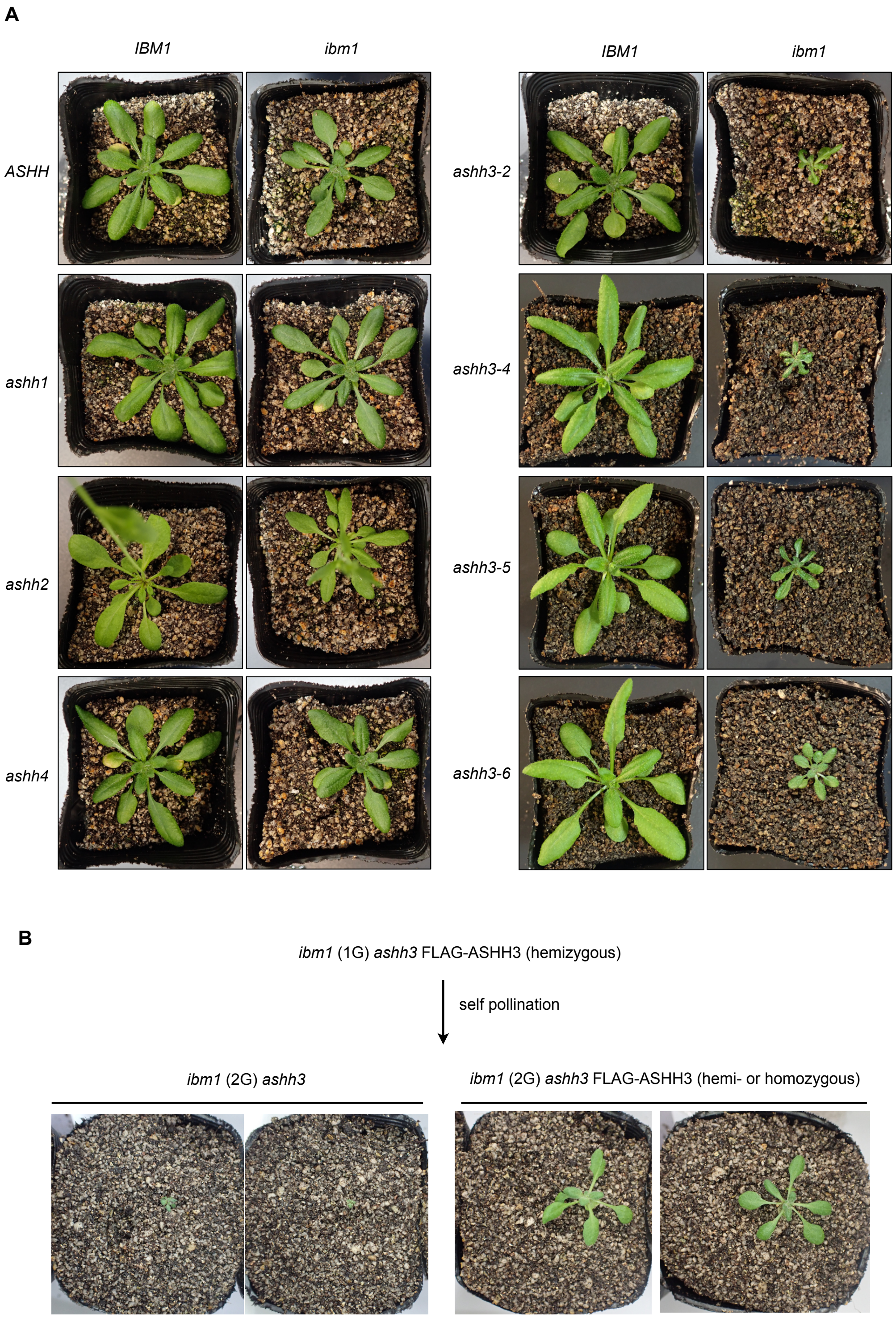

Fig. S4. Effect of *ashh* mutations on *ibm1* phenotype. (**A**) Four-week-old plants with combinatorial mutations of *ibm1* and *ashh1/2/3/4*. The gene names in capital letters and lower letters indicate wild-type allele and loss of function allele, respectively. *ashh3-2* allele is the same allele as Fig. 2B but the different individuals. *ashh3-4/5/6* are other alleles with independent T-DNA insertion events (see methods for material information). (**B**) Three-week-old plants of 2nd generation (2G) *ibm1 ashh3* double mutant with or without FLAG-ASHH3 transgene.

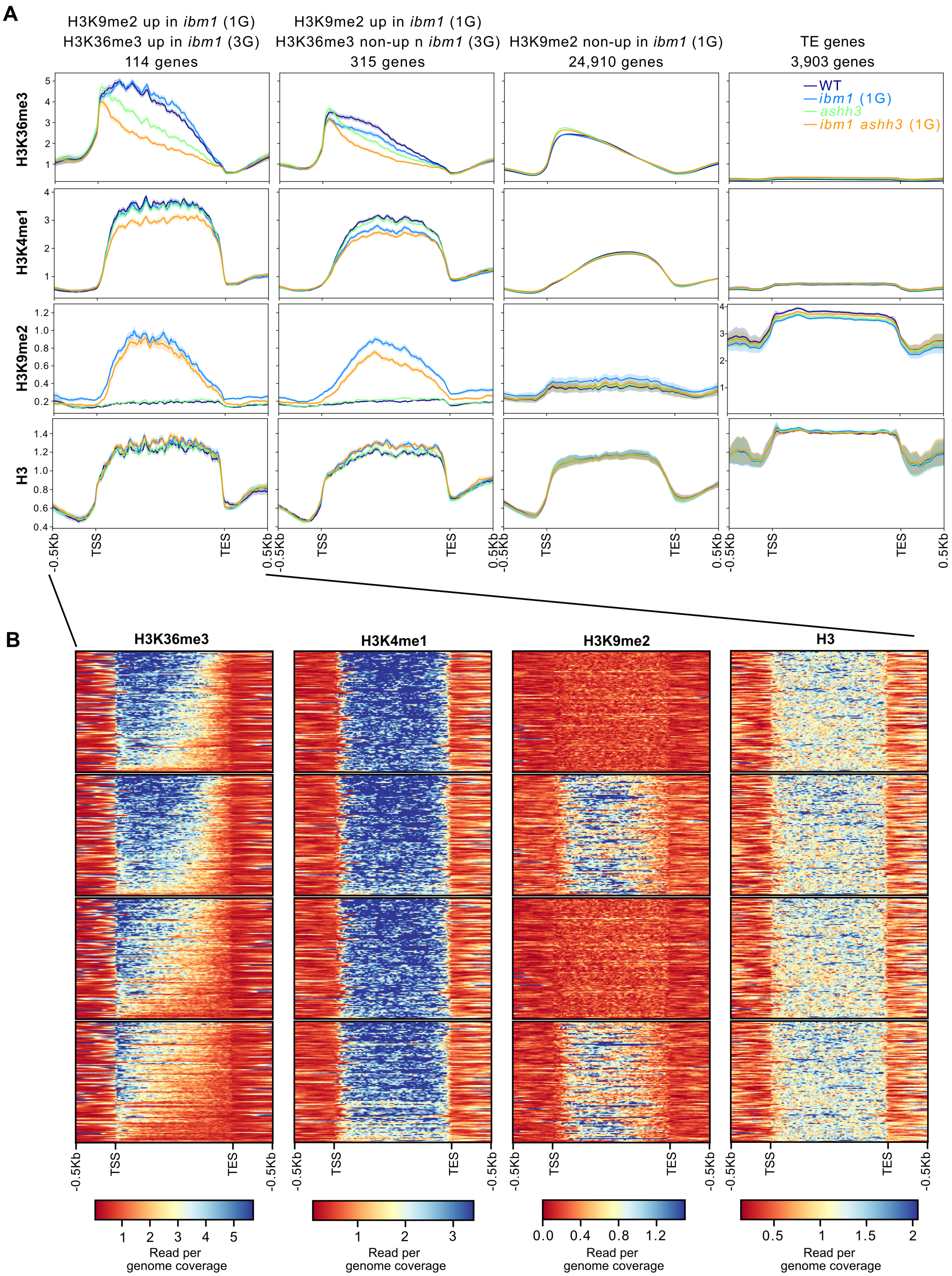

Fig. S5. Histone modification patterns in *ibm1* and *ibm1 ashh3*. (**A**) Intragenic patterns of H3K36me3, H3K4me1, H3K9me2, and H3 in *ibm1* (1G) and *ashh3* around genes categorized by H3K9me2 and H3K36me3 changes in *ibm1*, and TE genes. The y-axis represents the RPGC. (**B**) Heatmaps of H3K36me3, H3K4me1, H3K9me2, and H3 in *ibm1* (1G) and *ashh3* around 74 genes that are increased in H3K9me2 and H3K36me3 levels in *ibm1* (1G).

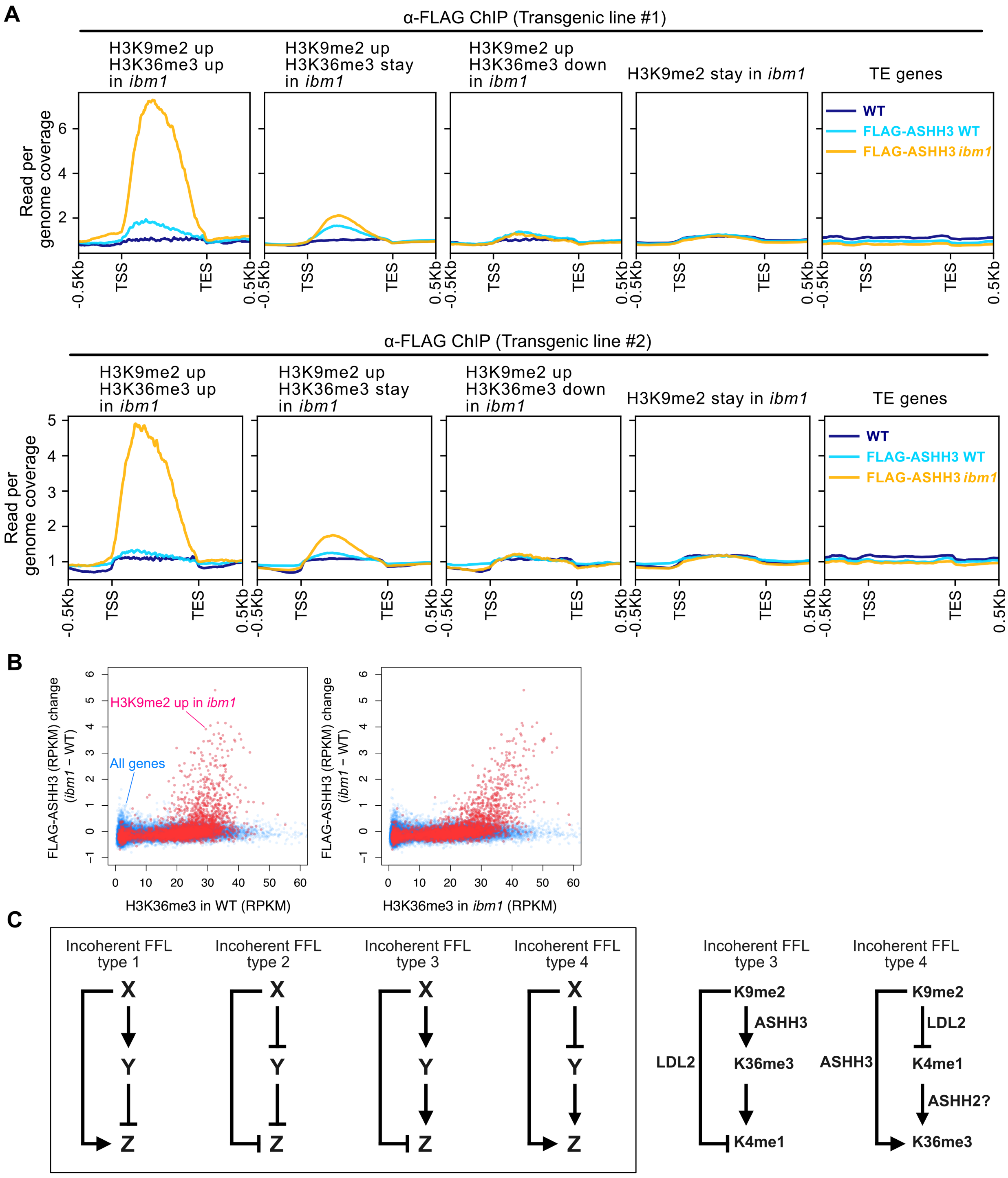

Fig. S6. ASHH3 localization and incoherent feedforward loops. (**A**) Intragenic patterns of FLAG-ASHH3 around genes categorized by H3K9me2 and H3K36me3 changes in *ibm1*, and TE genes. WT is used as the non-transgenic negative control. Two independent FLAG-ASHH3 transgenic lines were analyzed both in WT and *ibm1* background. (**B**) The relationship between H3K36me3 level in WT (left) and *ibm1* (right), and ASHH3 hyper-accumulation in *ibm1*. (**C**) Four types of incoherent FFLs (*22*) in the box and two incoherent FFLs revealed in this study (right).

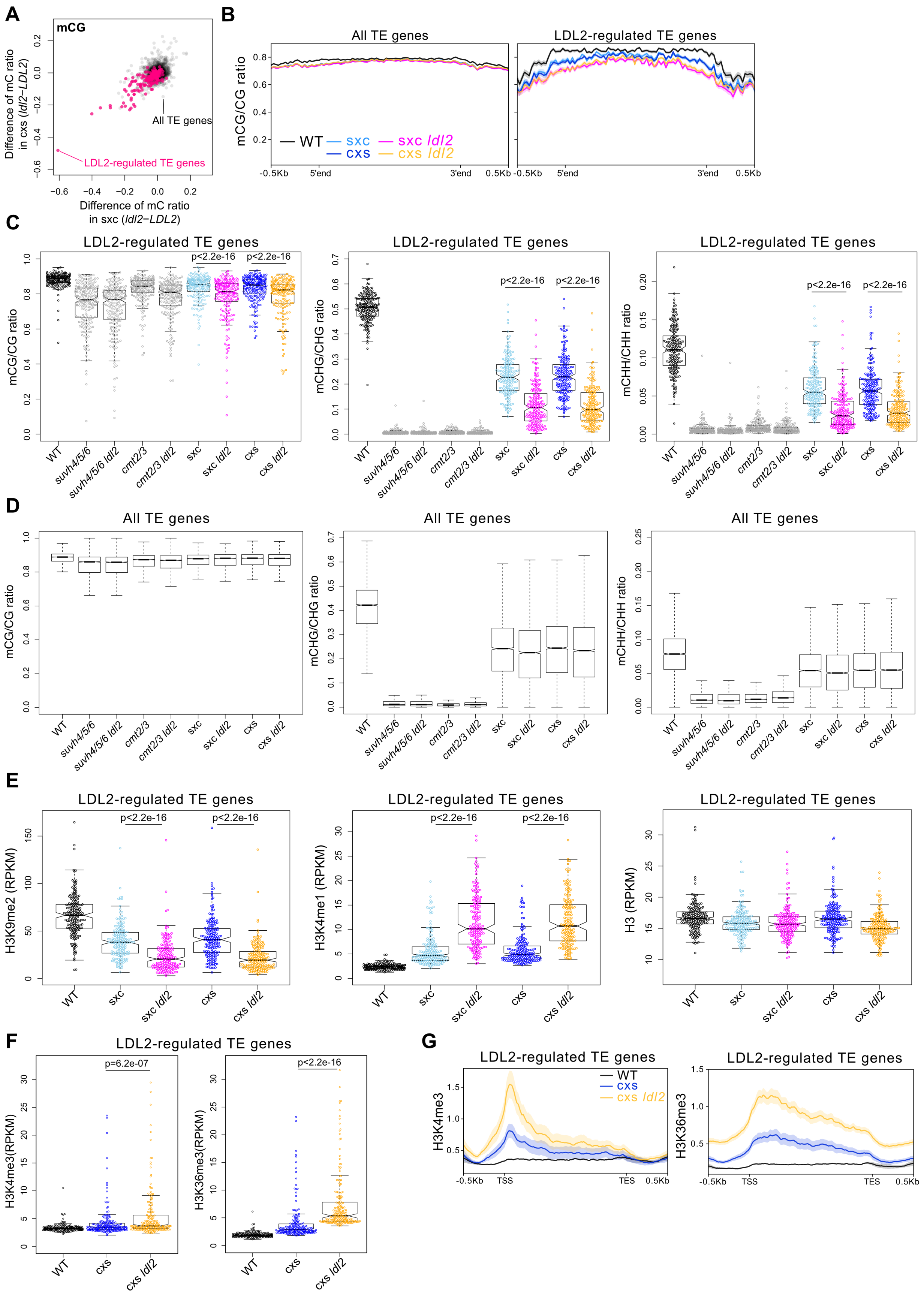
A
B
C
D
E
F
G
mCG
Difference of mC ratio in cxs (ldl2−LDL2)
Difference of mC ratio in sxc (ldl2−LDL2)
All TE genes
LDL2-regulated TE genes
mCG/CG ratio
WT
sxc
cxs
sxc ldl2
cxs ldl2
-0.5Kb
5'end
3'end
0.5Kb
mCHG/CHG ratio
mCHH/CHH ratio
p<2.2e-16
suvh4/5/6
suvh4/5/6 ldl2
cmt2/3
cmt2/3 ldl2
H3K9me2 (RPKM)
H3K4me1 (RPKM)
H3 (RPKM)
p=6.2e-07
H3K4me3(RPKM)
H3K36me3(RPKM)
H3K4me3
H3K36me3
TSS
TES

Fig. S7. LDL2-regulated establishment of TE silencing. (**A**) Effects of *ldl2* mutation on mCG in sxc (x-axis) and cxs (y-axis). Each dot represents each TE gene (n= 3,728) and red dots represent "LDL2-regulated TE genes" (n = 194), which show lower mCHG in sxc *ldl2* and cxs *ldl2* than in sxc *LDL2* and cxs *LDL2*, respectively (Fig. 3C). Averages of biological replicates are plotted. (**B**) Averaged profiles of mCG in WT and four F1 lines around all TE genes (left) and LDL2-regulated TE genes (right). (**C**) and (**D**) mCG (left), mCHG (middle), and mCHH (right) levels (ratios of methylated to all cytosines in each context) of LDL2-regulated TE genes (C) and all TE genes (D) in WT, parental mutant plants, and F1 plants. Each circle represents each TE gene. In the boxplots, the center line corresponds to the median, the notch represents the 95% confidence interval of the median, the upper and lower limits of the box correspond to the upper and lower quartiles, and the whiskers indicate the data range within 1.5x of the IQR. The *p*-values are based on paired *t*-tests. (**E**) H3K9me2 (left), H3K4me1 (middle), and H3 (right) levels of LDL2-regulated TE genes in WT and F1 plants. The *p*-values are based on paired *t*-tests. (**F**) H3K4me3 (left) and H3K36me3 (right) levels of LDL2-regulated TE genes in WT and F1 plants. The *p*-values are based on paired *t*-tests. (**G**) Averaged profiles of H3K4me3 (left) and H3K36me3 (right) in WT and F1 lines around LDL2-regulated TE genes. The y-axis represents the RPGC.

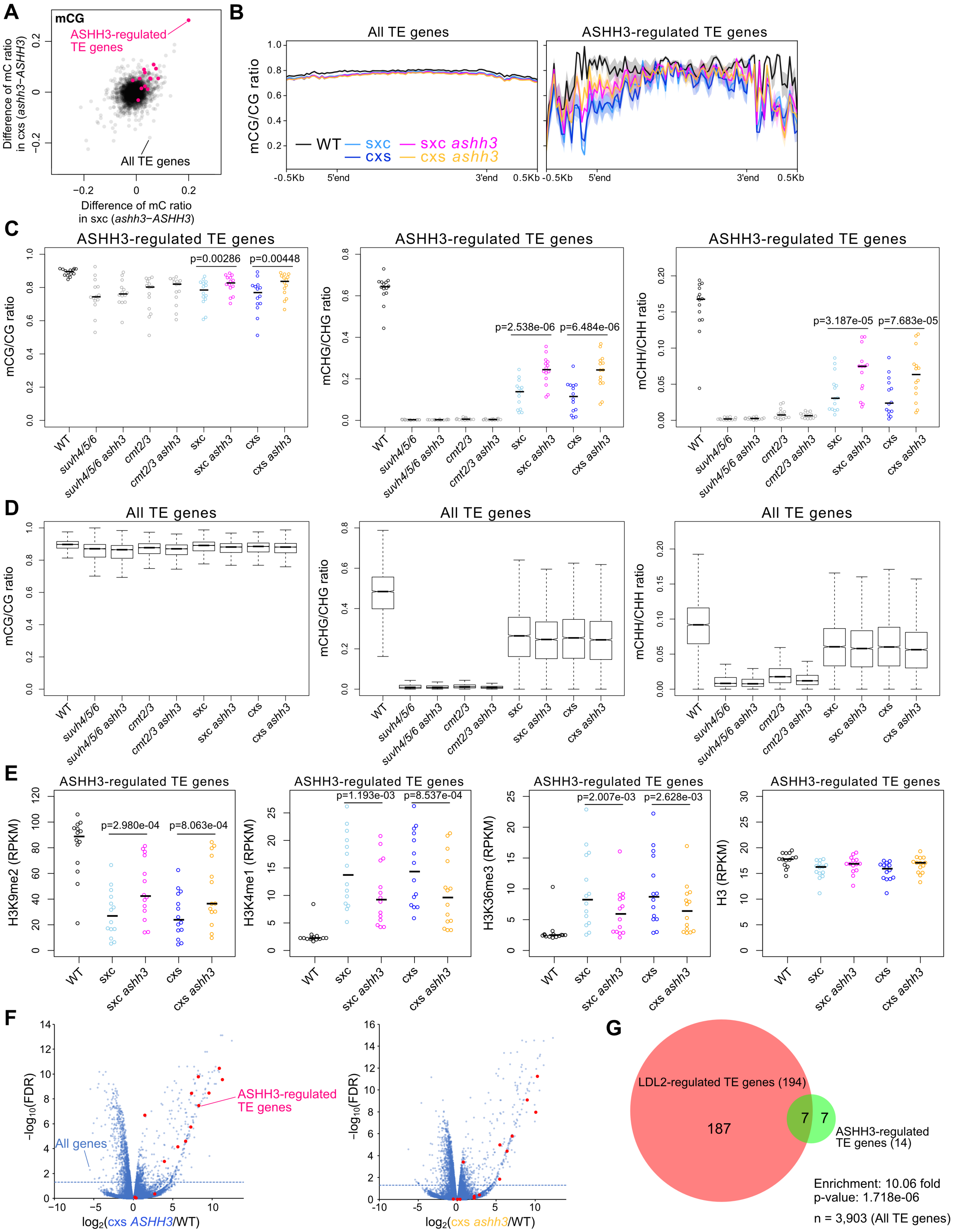
A
mCG
ASHH3-regulated TE genes
All TE genes
Difference of mC ratio in cxs (ashh3−ASHH3)
Difference of mC ratio in sxc (ashh3−ASHH3)
B
All TE genes
ASHH3-regulated TE genes
mCG/CG ratio
WT
sxc
cxs
sxc ashh3
cxs ashh3
-0.5Kb
5'end
3'end
0.5Kb
C
ASHH3-regulated TE genes
mCG/CG ratio
p=0.00286
p=0.00448
mCHG/CHG ratio
p=2.538e-06
p=6.484e-06
mCHH/CHH ratio
p=3.187e-05
p=7.683e-05
WT
suvh4/5/6
suvh4/5/6 ashh3
cmt2/3
cmt2/3 ashh3
sxc
sxc ashh3
cxs
cxs ashh3
D
All TE genes
mCG/CG ratio
mCHG/CHG ratio
mCHH/CHH ratio
E
ASHH3-regulated TE genes
H3K9me2 (RPKM)
p=2.980e-04
p=8.063e-04
H3K4me1 (RPKM)
p=1.193e-03
p=8.537e-04
H3K36me3 (RPKM)
p=2.007e-03
p=2.628e-03
H3 (RPKM)
F
−log10(FDR)
All genes
ASHH3-regulated TE genes
log2(cxs ASHH3/WT)
log2(cxs ashh3/WT)
G
LDL2-regulated TE genes (194)
187
7
7
ASHH3-regulated TE genes (14)
Enrichment: 10.06 fold
p-value: 1.718e-06
n = 3,903 (All TE genes)

Fig. S8. Effect of *ashh3* on the establishment of TE silencing. (**A**) Effects of *ashh3* mutation on mCG in sxc (x-axis) and cxs (y-axis). Each dot represents each TE gene (n= 3,551) and red dots represent "ASHH3-regulated TE genes" (n = 14), which show higher mCHG in sxc *ashh3* and cxs *ashh3* than in sxc *ASHH3* and cxs *ASHH3*, respectively (Fig. 4B). Averages of biological replicates are plotted. (**B**) Averaged profiles of mCG in WT and four F1 lines around all TE genes (left) and ASHH3-regulated TE genes (right). (**C**) and (**D**) mCG (left), mCHG (middle), and mCHH (right) levels (ratios of methylated to all cytosines in each context) of ASHH3-regulated TE genes (C) and all TE genes (D) in WT, parental mutant plants, and F1 plants. In (C), each circle represents each TE gene, and the horizontal bars indicate the medians. The *p*-values are based on paired *t*-tests. In the boxplots (**D**), the center line corresponds to the median, the notch represents the 95% confidence interval of the median, the upper and lower limits of the box correspond to the upper and lower quartiles, and the whiskers indicate the data range within 1.5x of the IQR. (**E**) H3K9me2, H3K4me1, H3K36me3, and H3 levels of ASHH3-regulated TE genes in WT and F1 plants. The *p*-values are based on paired *t*-tests. (**F**) Volcano plots of mRNA-seq comparing TPM of WT and cxs *ASHH3* (left), and WT and cxs *ashh3* (right). Blue dotted lines represent FDR = 0.05. Blue dots represent all genes including protein-coding genes and TE genes (n = 33,603), and red dots represent ASHH3-regulated TE genes. (**G**) Venn diagram showing the overlap between LDL2-regulated TE genes and ASHH3-regulated TE genes. The *p*-value is based on a hypergeometric test.

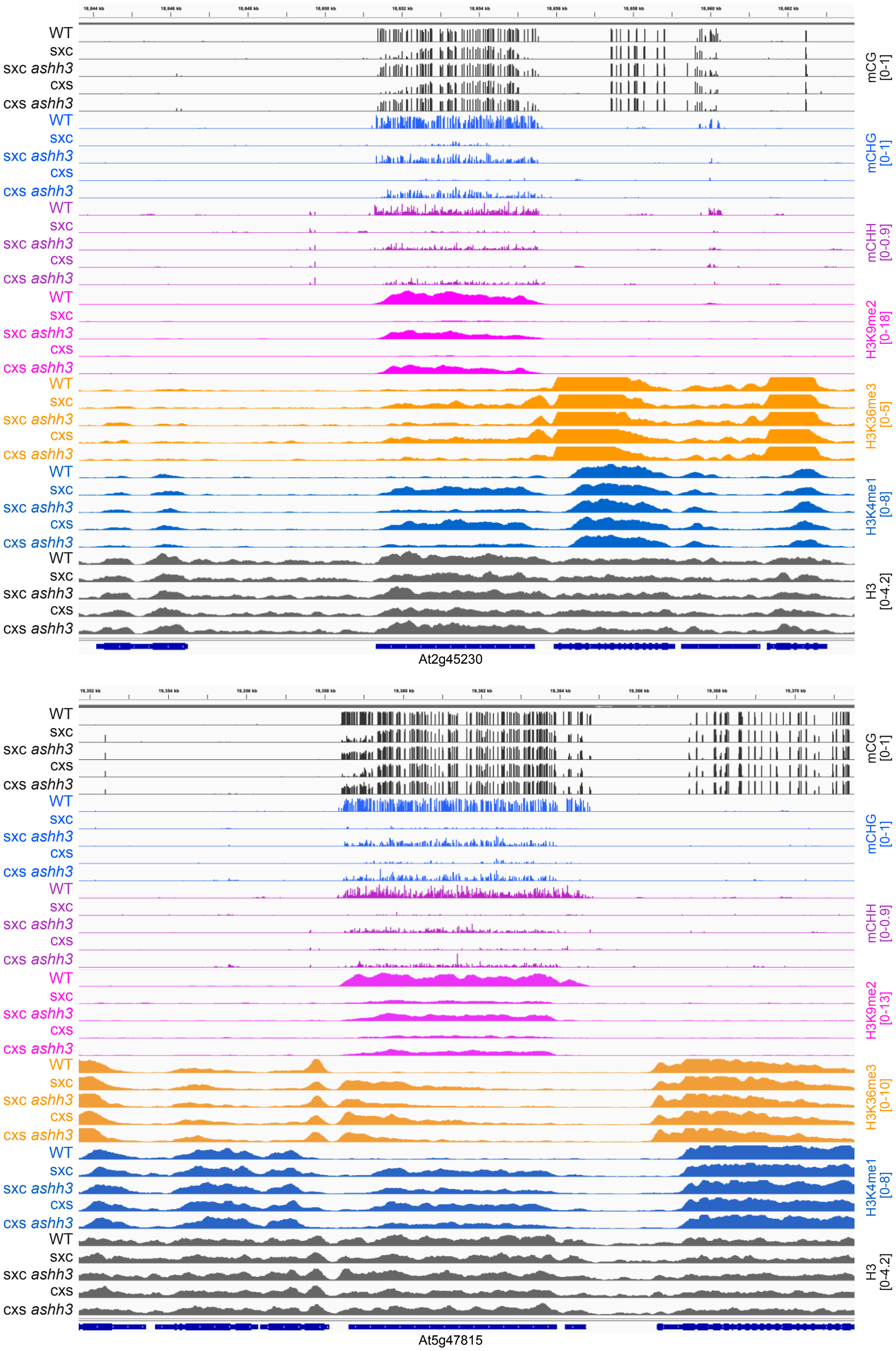

Fig. S9. TEs anti-silenced by ASHH3. Browser views showing DNA methylation in CG, CHG, CHH contexts, H3K9me2, H3K36me3, H3K4me1, and H3 around two ASHH3-regulated TE genes, At2g45230 (top) and At5g47815 (bottom).

Data S1. (separate file)
The list of genes analyzed in Figures.
